# Treatment and Outcome of Patients With Coronary Artery Ectasia: Current Evidence and Novel Opportunities for an Old Dilemma

**DOI:** 10.3389/fcvm.2021.805727

**Published:** 2022-02-04

**Authors:** Luca Esposito, Marco Di Maio, Angelo Silverio, Francesco Paolo Cancro, Michele Bellino, Tiziana Attisano, Fabio Felice Tarantino, Giovanni Esposito, Carmine Vecchione, Gennaro Galasso, Cesare Baldi

**Affiliations:** ^1^Department of Medicine, Surgery and Dentistry, University of Salerno, Salerno, Italy; ^2^Division of Interventional Cardiology, Cardiovascular and Thoracic Department, San Giovanni di Dio e Ruggi, Salerno, Italy; ^3^Cath Lab Unit, Cardiovascular Department, Morgagni Hospital, Vecchiazzano-Forlì, Italy; ^4^Department of Advanced Biomedical Sciences, Federico II University of Naples, Naples, Italy; ^5^Vascular Pathophysiology Unit, Istituto di Ricovero e Cura a Carattere Scientifico (IRCCS) Neuromed, Pozzilli, Italy

**Keywords:** coronary artery ectasia, percutaneous coronary intervention, coronary artery disease, acute coronary syndrome, antithrombotic therapy

## Abstract

Coronary artery ectasia (CAE) is defined as a diffuse or focal dilation of an epicardial coronary artery, which diameter exceeds by at least 1. 5 times the normal adjacent segment. The term ectasia refers to a diffuse dilation, involving more than 50% of the length of the vessel, while the term aneurysm defines a focal vessel dilation. CAE is a relatively uncommon angiographic finding and its prevalence ranges between 0.3 and 5% of patients undergoing coronary angiography. Although its pathophysiology is still unclear, atherosclerosis seems to be the underlying mechanism in most cases. The prognostic role of CAE is also controversial, but previous studies reported a high risk of cardiovascular events and mortality in these patients after percutaneous coronary intervention. Despite the availability of different options for the interventional management of patients with CAE, including covered stent implantation and stent-assisted coil embolization, there is no one standard approach, as therapy is tailored to the individual patient. The abnormal coronary dilation, often associated with high thrombus burden in the setting of acute coronary syndromes, makes the interventional treatment of CAE patients challenging and often complicated by distal thrombus embolization and stent malapposition. Moreover, the optimal antithrombotic therapy is debated and includes dual antiplatelet therapy, anticoagulation, or a combination of them. In this review we aimed to provide an overview of the pathophysiology, classification, clinical presentation, natural history, and management of patients with CAE, with a focus on the challenges for both clinical and interventional cardiologists in daily clinical practice.

## Introduction

Coronary artery ectasia (CAE) is defined as a diffuse or focal dilation of an epicardial coronary artery, with a diameter that exceeds of at least 1.5 times the normal adjacent segment. It is described in up to 5% of all comers' patients undergoing coronary angiography, but with a considerable variability in relation to the patients' clinical presentation and the definition adopted for CAE (1, 2). While atherosclerosis seems to be the most frequent etiopathogenetic mechanism, other possible causes include systemic inflammatory vasculitis, connective tissue disorders, genetic diseases, infections, and iatrogenic injury following percutaneous coronary intervention (PCI) (3, 4). CAE shows a wide spectrum of clinical manifestations, ranging from incidental findings in asymptomatic patients, to effort angina, exercise-induced ischemia, and acute coronary syndrome (ACS) (5, 6). Although the prognosis of CAE still represents a matter of debate, several studies reported a high risk of adverse events at long-term follow up in patients with myocardial infarction (MI) and angiographic evidence of CAE (7–9). The treatment of patients with CAE constitutes an unsolved problem both for clinical and interventional cardiologists since each therapeutic option offers its own advantages and drawbacks in this setting. The abnormal coronary dilatation and flow disturbances, often associated with high thrombus burden in patients with MI, advocate more potent and prolonged antithrombotic therapies. However, in absence of robust large-scale data, the pharmacological treatment is not standardized yet and still relies on the choice of the clinicians based on their own experience.

The aim of the present review is to outline the classification, etiopathogenesis, clinical presentation and diagnostic assessment of CAE, with a specific focus on management strategies and long-term outcome.

## Definition and Classification

**Figure 1:**
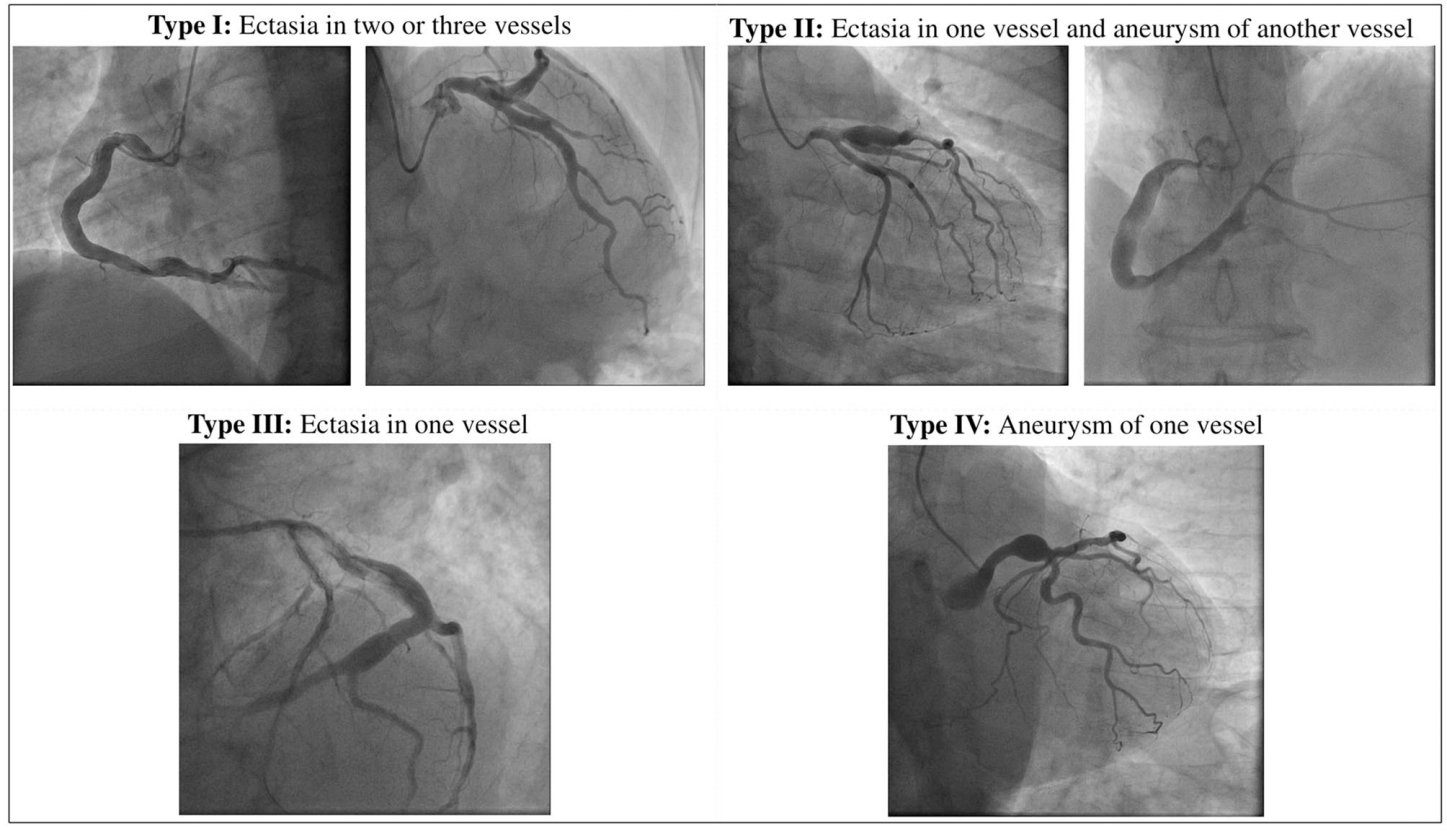
Anatomical definition of CAE according to Markis classification.

To date the definition of CAE remains uncertain: the lexical variability prevents to accumulate the data for robust scientific evidence on this issue. The mainstream of the literature directs the nomenclature on the basic distinction between the terms ectasia and aneurysm that, although sometimes used as synonyms, represent two different phenotypes of the disease: while the first one identifies a diffuse dilatation that involves > of 50% of the length of the vessel, the term coronary artery aneurysm (CAA) refers a focal dilatation (10). CAAs are also subclassified into saccular if the transverse diameter exceeds the longitudinal diameter, and fusiform in the opposite case. Giant CAAs are defined instead as a dilatation with a diameter >20 mm or if the diameter exceeds the reference vessel diameter by >4 times in adults, while in children if the diameter is >8 mm (11, 12). Moreover, according to the integrity of the vessel architecture, CAAs can be also divided into true or pseudoaneurysms. While true CAAs involve the three layers of vessel tunica, pseudoaneurysms are single- or double-layer dilatations that occurs after the disruption of the media and external elastic membrane, usually caused by blunt chest trauma or mechanical damage during PCI (13–15). A topographical classification of CAE was proposed by Markis et al. Briefly, CAE is divided into 4 anatomical phenotypes according to its extension in the coronary tree: diffuse ectasia of two or three vessels is classified as type I, diffuse ectasia in one vessel and focal dilatation in another vessel as type II, diffuse ectasia of one vessel only and focal aneurysm as type III and IV, respectively (16). Angiographical subtypes according to Markis classification are shown in Figure 1.

## Epidemiology

CAE is a relatively uncommon angiographic finding, with a prevalence ranging from 0.3 to 5% of all comers' patients undergoing coronary angiography (1, 2, 17). The wide variability in the reported prevalence has several reasons, including the lack of homogeneity in the adopted definition of CAE, a certain inter-operator variability in the angiographic evaluation, geographical discrepancies, and the different clinical presentations of the patients included in the studies. Indeed, an even higher prevalence of CAE was reported in specific clinical settings, reaching up to 9% in some cohorts of patients with ST-segment elevation MI (STEMI) (7). Nevertheless, since studies include only patients undergoing coronary angiography and/or presenting with ACS, it is reasonable to think that the real prevalence in the general population may be overestimated.

Several reports showed a gender predominance with a high prevalence of CAE in men (1, 18, 19). Hypertension, smoking and dyslipidemia, including familial hypercholesterolemia, have also been associated with CAE (20–22). A higher risk of CAE was also reported among cocaine abusers (23). Nevertheless, in contrast with coronary artery disease (CAD), CAE has an inverse association with diabetes mellitus (24, 25). Since diabetes mellitus promote negative remodeling of the artery wall, the impairment of compensatory vessel enlargement might explain the lower prevalence of CAE in diabetic patients (26).

Right coronary artery (RCA) is the most frequently involved, followed by left anterior descending (LAD), left circumflex (LCx), and less frequently left main (LM) (26).

CAE has been frequently reported in patients with aneurysms in other vascular beds, such as ascending and abdominal aorta and pulmonary arteries (27). Moreover, Meindl et al. found that CAE was a common finding in patients with bicuspid aortic valve (BAV), with or without aneurysms of the ascending aorta (28).

## Etiopathogenesis and Pathophysiology

Although the etiopathogenesis of CAE is not fully understood, atherosclerosis seems to be the most frequent cause in adults (18). This hypothesis is supported by the frequent coexistence with CAD and by the observation of common histopathological findings, like lipid deposition and hyalinization, destruction and reduction of the medial elastic fibers and disruption of the internal and external elastic lamina (16, 29, 30). These observations lead to the assumption of CAE as a variant of CAD (31).

However, CAE shows some other histopathological features that are not usually observed in patients with CAD, like the relative preservation of the intima and the extensive loss of musculoelastic components of the media, which is thought to be one of the central mechanisms in the pathogenesis of CAE (32, 33). Indeed, an exaggerated activity of metalloproteinases (MMP) has been demonstrated in CAE (34, 35). The extreme proteolysis of extracellular matrix (ECM) weakens the vessel architecture, increase the wall stress, and reduce vessel tolerance to blood flow, thus predisposing to the progressive dilatation of the artery. Moreover, chronic inflammation further contributes to arterial wall damage and dilatation, as demonstrated by the correlation between high levels of inflammatory markers and the presence and the severity of CAE (36–38). However, while inflammation represents one of the major features of the pathophysiology and natural history of atherosclerosis, recent findings demonstrated an increased inflammatory status in patients with CAE compared to those with CAD alone, with significantly higher levels of circulating cytokines (39). Therefore, it might be reductive to categorize CAE as a simple variant of CAD. CAE could rather be considered as the coronary manifestation of an aggressive phenotype of systemic vascular disease, pathogenetically linked to CAD in most cases, despite some morphological, pathophysiological, and clinical discrepancies with the typical atherosclerotic disease.

The coexistence with other conditions, such as BAV and aortic aneurysms, corroborates the conception of CAE as the manifestation of a systemic disease, in which individual genetical susceptibility might be one of the contributing factors (17, 28).

Systemic inflammatory diseases, infections and connective tissue disorders are other possible causes of CAE (12, 40, 41). Kawasaki disease (KD), a systemic vasculitis with coronary tropism, is the most common etiology of CAE in childhood (42). KD-associated CAE occurs in up to 23% of untreated patients and has distinctive anatomical features, such as the preferential involvement of proximal rather than distal segments, and the development of focal rather than diffuse coronary dilatations (12, 43). From a pathophysiological perspective, an increased activity of MMPs has been observed also in patients with KD and coronary vasculitis, emerging like a common underlying mechanism that reconciliates KD with other etiologies of CAE, including CAD-associated forms (44, 45).

CAE has also been reported among cocaine users and its pathogenesis may be related to severe hypertension episodes and to the direct endothelial damage caused by drug-induced vasoconstriction (23).

CAAs or pseudoaneurysms are rare but potential complications of PCI, especially following brachytherapy, atherectomy, or drug-eluting stent (DES) implantation (46–48). Residual dissection and deep arterial wall injury caused by oversized balloons or stents, high-pressure balloon inflations and atherectomy, can result in coronary dilatation due to direct vessel injury and secondary healing process (49). Additionally, the release of anti-proliferative drugs after DES implantation, while dramatically reducing the risk of in-stent restenosis (ISR), might potentially cause CAAs due to delayed re-endothelization, impaired healing after vessel injury, inflammatory changes of the medial wall and hypersensitivity reaction to the polymer carrying the drug (4, 50, 51). However, most of the reported cases refers to the first-generation DES era: the progressive development of new technologies, including the introduction of thinner struts, bioabsorbable or polymer-free DES with improved biocompatibility, and new anti-proliferative drugs, might justify a reduction in the incidence of this complication (52). There are also several reports of CAAs development following bioabsorbable vascular scaffold (BVS) implantation: gradual scaffold degradation, strut discontinuity, and consequential displacement of the BVS might be possible underlying mechanisms (53–55).

A distinct setting of aneurysmatic disease is represented by aortocoronary saphenous vein graft aneurysms (SVGAs). SVGAs are a rare and late complication of coronary artery bypass grafting (CABG), usually occurring after ~15 years from surgery. As for CAE, etiopathogenesis is not fully understood and several mechanisms have been proposed, including structural deterioration caused by graft atherosclerosis, technical issues related to surgical manipulation and adaptation of vein graft to higher arterial pressure with vessel wall weakening and dilatation. SVGAs usually progress over time, often reaching large diameter, and have a high incidence of life-threatening mechanical complications, such as compression of adjacent structures, fistulous communications, and rupture (56, 57).

**Figure 2:**
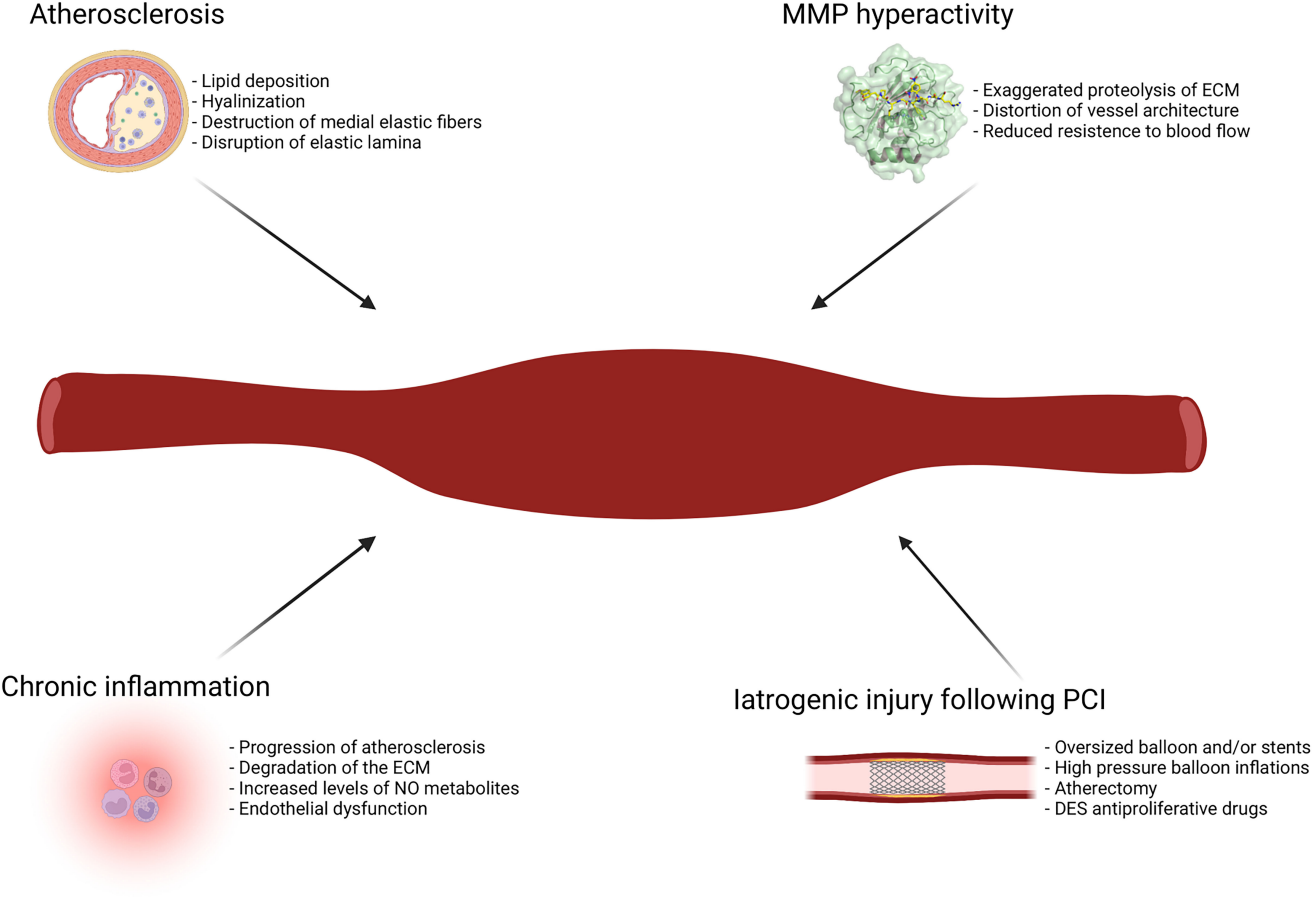
Main etiopathogenetic mechanisms of CAE. DES, drug-eluting stent; ECM, extracellular matrix; MMP, metalloproteinases; NO, nitric oxide; PCI, percutaneous coronary intervention.

Figure 2 illustrates the main etiopathogenetic mechanisms of CAE.

## Clinical Presentation and Diagnostic Assessment

**Figure 3:**
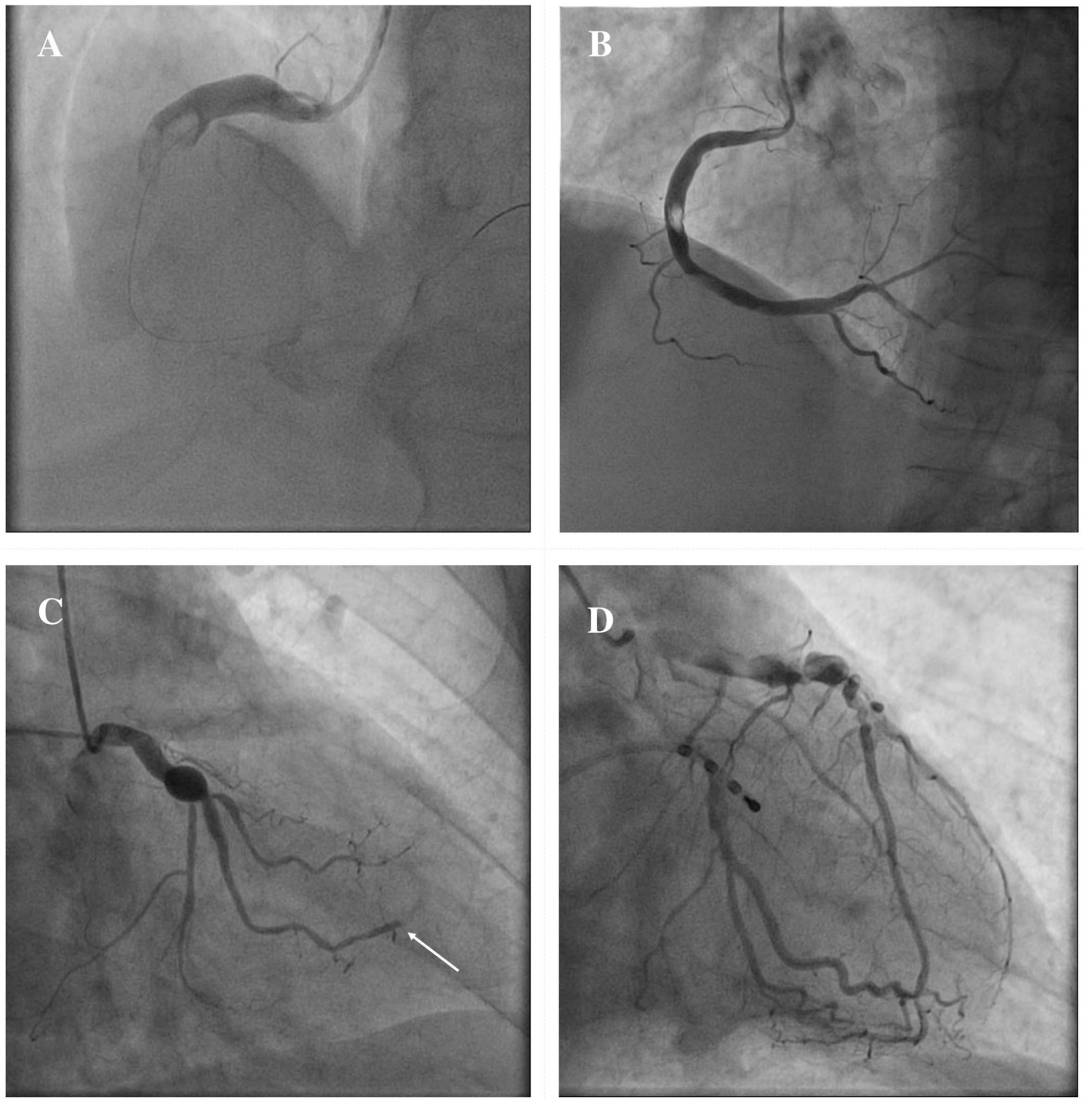
Mechanisms of ACS in patients with CAE. ACS in patients with CAE can occur through different pathophysiological mechanisms. Catastrophic plaque rupture resulting in acute vessel occlusion due to a huge thrombotic burden untreatable despite a timely primary PCI **(A)**. Relevant filling defect due to endoluminal thrombus without significant underlying atherosclerotic plaque, to be ascribed to flow disturbances in an entirely ectatic RCA **(B)**. Abrupt flow occlusion of the distal segment in a marginal branch (arrow) due to the embolization of clot fragments coming from a proximal saccular CAA of the LCx **(C)**. Ectatic LAD showing images of advanced flow disturbances angiographical pattern, that suggest the relationship between impaired blood progression and myocardial ischemia **(D)**. ACS, Acute Coronary Syndrome; CAA, coronary artery aneurysm; CAE, Coronary Artery Ectasia; LAD, left anterior descending; LCx, left circumflex; PCI, percutaneous coronary intervention; RCA, right coronary artery.

The clinical manifestations of CAE are heterogeneous, and often it is occasionally recognized during coronary angiography or computed tomography (CT). However, CAE may become clinically overt through different possible scenarios, including ACS, effort angina, exercise-induced ischemia, microvascular dysfunction, compression of adjacent cardiac or non-cardiac structures and the tragical, albeit rare, complication of rupture with acute cardiac tamponade (5, 58–60). In patients with CAE, there are several possible mechanisms leading to the clinical event of an ACS: (1) atherosclerotic plaque instability with high thrombus burden; (2) endoluminal thrombosis due to flow disturbances and blood stasis, in absence of underlying atherosclerotic lesions; (3) distal embolization of thrombotic material; (4) impairment of myocardial perfusion related to the severe slow flow, which can be clinically expressed either as ACS or effort angina. Figure 3 illustrates the main mechanisms of ACS in patients with CAE.

The gold standard for the diagnostic assessment of CAE remains coronary angiography: the typical angiographical features are delayed antegrade contrast filling, segmental back flow, and local deposition of dye in the dilated coronary segment, all of them able to depict the severe flow disturbances of CAE. However, slow flow and blood stasis might complicate the angiographic assessment of CAE, especially when evaluating the true size of the vessel or the presence of thrombotic material. IVUS is useful to better assess vessel wall architecture and to distinguish between true aneurysm, pseudoaneurysm, normal segments with adjacent stenosis or complex plaques angiographically mimicking CAAs (13): this differential diagnosis is crucial for prognostic stratification and therapeutic guidance, due to the high risk of ACS associated with the presence of an ulcerated or ruptured atherosclerotic plaque. Moreover, IVUS might be helpful to precisely estimate the minimal lumen area and the percentage of the stenosis, to evaluate the thrombotic and calcific burden of the lesion, and to guide stent sizing and implantation when PCI is planned (61).

Optical coherence tomography (OCT) is another possible tool in the invasive assessment of CAE. Compared to IVUS, OCT has greater axial and spatial resolution that might be helpful in CAE to assess atherosclerotic plaque features, thrombus burden and mechanisms of PCI failure. However, its application in large vessel is limited by the low penetration depth, with loss of image definition as the distance of the anatomical structures from the lens increases (62).

Other technologies are available for the interventional cardiologist in the toolbox of CAE investigation, but they play a minor role. CT may be helpful in the non-invasive assessment of CAE, increasing the prevalence of incidentally found CAE, which was described as a rare finding on coronary angiography in previous decades (2, 63). CT provides important information on CAE anatomical features, such as shape, maximum diameter and presence of concomitant stenosis, avoiding the pitfalls of coronary angiography (64). Furthermore, CT allows a three-dimensional reconstruction of the coronary tree that might clarify anatomical and functional relations with other adjacent structures (i.e., fistulous communications with cardiac or non-cardiac chambers) (65, 66).

**Figure 4:**
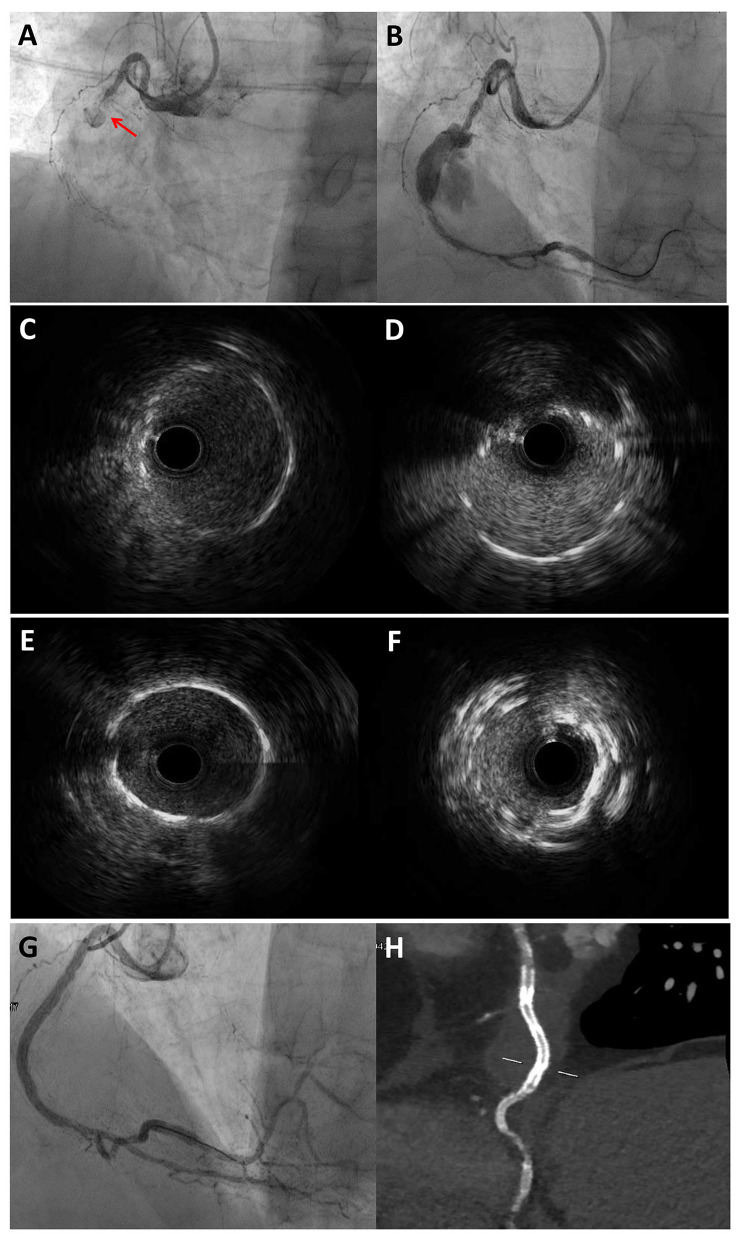
Multimodality imaging approach for diagnosis and PCI guidance in a STEMI patient with angiographic evidence of CAE. 77-year-old man admitted for STEMI, who underwent emergent coronary angiography. Coronary angiography showed an occlusion of the mid RCA due to stent thrombosis **(A)** and a CAA distal to the occlusion site, which was visible after guidewire crossing and thrombectomy **(B)**. IVUS evaluation showed the previously implanted stents at the proximal **(C)** and distal **(D)** necks of the CAA. The PCI strategy consisted of 48 mm EES implantation bridging the proximal and distal CAA necks to create a supporting platform for the deployment of two overlapping covered stents. **(E)** and **(F)** show the proximal and distal edges of the EES assessed by IVUS. Coronary angiography showed an optimal sealing of the CAA after the implantation of two overlapping 3.5 × 24 mm single-layer PTFE covered stents (BeGraft, Bentley InnoMed, Hechingen, Germany) **(G)**, which was confirmed by CCTA after the procedure **(H)**. ^*^Courtesy of Dr. Iacopo Muraca. CAA, coronary artery aneurysm; CCTA, coronary computed tomography angiography; EES, everolimus-eluting stent; IVUS, intravascular ultrasound; PTFE, polytetrafluoroethylene; RCA, right coronary artery; STEMI, ST-segment elevation myocardial infarction.

Figure 4 shows the role of a multimodality imaging approach in a patient with STEMI and angiographic evidence of CAE.

Magnetic resonance angiography (MRA) is another possible tool in the assessment of CAE, especially in patients with contraindication for other diagnostic techniques. MRA provides important anatomical and functional information, especially in large proximal vessels, while its resolution decreases in smaller and distal segments (67, 68).

Transthoracic and transesophageal echocardiography have a role in the evaluation of patients with CAE, especially in children with KD and large CAAs of the proximal LM and RCA. Echocardiography is useful to assess the location of the CAA and the presence of intraluminal thrombi. Given its non-invasive nature, it is an ideal method for long-term follow up in adulthood of patients with history of KD (43).

## Natural History and Prognostic Stratification

The long-term outcome of patients with CAE has been poorly investigated. Although flow disturbances, enhanced thrombogenicity and high risk of procedural drawbacks during PCI would be perceived as associated to unfavorable prognosis, literature does not provide conclusive evidence on the clinical outcome of patients with CAE. Krüger et al. demonstrated the occurrence of angina and exercise-induced ischemia in patients with isolated CAE and non-obstructive CAD, suggesting that flow turbulences might potentially impair myocardial perfusion even in absence of coexisting significant stenoses (5). However, the prognostic role of isolated CAE still needs to be clarified. Furthermore, it is not clear whether CAE confers additional risk in patients with coexisting CAD. In a large series from the CASS study, the presence of CAE did not affect the adjusted 5-year survival of patients with CAD (1). Similar findings were reported by Demopoulos et al. with no evidence of additional risk of adverse events in patients with CAE (18). In opposition, Baman et al. found that CAE had an independent adverse effect on long term mortality, without significant differences between patients with and without CAD (69). Several reasons might explain the inconsistency between these studies, such as the small number of patients included, the lack of an adequate follow-up and the heterogeneity in the adopted definition of CAE.

Nonetheless, recent evidence demonstrated a high risk of cardiovascular events at long term in patients with ACS and angiographic evidence of CAE. The prognostic implications of CAE in the high-risk clinical setting of ACS might have several explanations. First, the presence of an ectatic infarct-related artery (EIRA), often associated with high complexity of the lesion and large thrombus burden, might affect *per se* the procedural success of PCI and the long-term clinical outcome of these patients. Bogana Shanmugam et al. compared 25 STEMI patients with an EIRA to a cohort of 80 non-EIRA STEMI patients and found that the EIRA group, despite similar in-hospital outcomes, had a higher incidence of long-term cardiovascular events, particularly driven by recurrent MI, unstable angina (UA), and need for surgical revascularization (70). Conversely, in a recent metanalysis of 6 observational studies on patients with STEMI treated with primary PCI, no difference in terms of mortality were reported between CAE and non-CAE patients, despite a higher thrombus burden and a lower post-procedural TIMI flow in the CAE group (71).

Similarly, Ipek et al. showed no difference in terms of in-hospital and 1 year mortality and revascularization between STEMI patients with and without EIRA undergoing primary PCI, despite a higher rate of no-reflow in the EIRA group (72). However, the unadjusted statistical analysis, the relatively small sample size, the paucity of adverse events, and the short-term follow-up time might have influenced these findings. In a study on 643 consecutive patients with STEMI treated with primary PCI, patients with EIRA were compared to a control group of non-EIRA and showed suboptimal procedural result of primary PCI with impaired epicardial flow and myocardial perfusion, larger thrombus burden, higher risk of distal embolization and lower ST-segment resolution and vascular collateral development. Moreover, the presence of an EIRA emerged as an independent predictor of adverse outcome (73). However, there are growing evidence that patients with ACS and angiographic evidence of CAE have a high risk of future adverse events at follow up, irrespective of the presence of an EIRA. In a single-center observational study conducted by our group on 534 patients with STEMI, we found that 154 CAE patients developed a higher risk of recurrent MI at long-term follow up compared to a propensity-weighted group of 380 non-CAE STEMI (7). However, no significant differences in all-cause death and cardiac death were reported between groups: probably a longer follow up and a larger number of patients might have yielded more conclusive results in terms of long-term mortality. Similarly, an observational study on 1,698 Japanese patients with acute MI showed a significantly higher risk of major cardiac events (defined as the composite of cardiac death and non-fatal MI) in 51 patients with CAE compared to a propensity-matched cohort of 1,647 patients without CAE (8). Furthermore, Wang et al. compared the long-term clinical outcome of 174 CAE patients with MI and 4,614 patients with MI and no evidence of CAE: at a median follow up of 4 years, CAE patients showed a significantly higher incidence of the composite of cardiac death, MI, stroke and repeated coronary revascularization; at multivariable analysis, CAE emerged as an independent predictor of recurrent cardiovascular events (74).

In accordance with these findings, Gunasekaran et al. assessed the prognostic significance of CAE anatomical extension in a retrospective study on 317 patients and found that a higher grade of CAE (Markis type I and II) was associated with a significantly higher risk of ACS at long term compared to lower grade of CAE (Markis III and IV), despite similar severity of underlying CAD. Moreover, patients with Thrombolysis In Myocardial Infarction (TIMI) flow <3 showed a higher occurrence of ACS at follow up compared to patients with normal epicardial flow, with further adverse impact on outcomes when associated with multivessel CAE (9). A cohort study on 595 Chinese patients with CAE, compared the clinical outcome of the two anatomical phenotypes of CAE, diffuse CAE, or ectasia, and focal CAE, or CAAs: patients with diffuse CAE, compared to the group with focal coronary dilations, showed a significantly higher incidence of major cardiovascular events, defined as cardiovascular death and non-fatal MI, at both propensity-matched and propensity-weighted analyses (75). These findings might indicate that a diffuse extension of CAE in the coronary tree results in a greater impairment of coronary flow and a higher risk of thrombotic and embolic events, thus affecting long-term clinical outcome of these patients.

Taking all these findings together, it is reasonable to consider CAE as an aggressive phenotype of coronary disease associated with a multifactorial risk of cardiovascular events at follow up: technical issues of PCI and the intrinsic complexity of the disease lead to the imbalance of coronary hemodynamics, susceptibility to local thrombotic disorders and adverse clinical outcome.

The main studies evaluating clinical outcome of patients with CAE are summarized in Table 1.

**Table 1:** Main studies evaluating clinical outcome in patients with CAE.

**References**	**No. of CAE patients**	**CAE phenotype**	**Clinical setting**	**Primary outcomes**	**Results**
Baldi et al. (7)	154	All CAE phenotypes	STEMI	Recurrence of MI compared with 380 controls at 1,218.3 ± 574.8 days of f/u	Higher recurrence of MI in CAE patients (HR 1.84; 95% CI 1.11–3.05; *p* = 0.017)
Will et al. (76)	81	All CAE phenotypes	STEMI NSTEMI CCS	MACE in patients with CAA treated with covered stent	The use of covered stents for elective treatment of CAA is effective and reasonably safe
Wang et al. (74)	174	All CAE phenotypes	STEMI NSTEMI	MACE compared with 4,614 non-CAE patients at a median f/u of 4 years (1–7)	CAE is associated with higher recurrence of MACE (HR 1.597; 95% CI 1.238–2.060; *p* < 0.001)
Khubber et al. (77)	458	Only CAAs	All comers patients undergoing ICA	MACCE in patients treated with medical therapy (230), PCI (52), or CABG (176) at a median f/u of 62 months (11–120)	Similar long-term outcomes in patients with CAA undergoing medical, percutaneous, and surgical management (OR 0.773; 0.526–1.136; *p* = 0.19)
Cai et al. (75)	595	All CAE phenotypes	All comers patients undergoing ICA	MACE during a median f/u of 87 months (72–104)	Higher risk of MACE in patients with diffuse CAE than focal CAE (HR 3.26, 95% CI 1.17–9.04, *p* = 0.023)
D'Ascenzo et al. (78)	585	Only CAAs	All comers patients undergoing ICA	Composite of MI, UA, and aneurysm thrombosis compared to 390 control patients at a median f/u of 3 years (1–7)	OAC decreases the composite endpoint (8.7 vs. 17.2%; *p* = 0.01), non-significant higher risk of bleeding
Nuñez-Gil et al. (6)	1,565	Only CAAs	All comers patients undergoing ICA	Composite of MI, UA, and aneurysm thrombosis compared with 380 control patients at a median f/u of 3 years (1–7)	OAC decreases the composite endpoint (8.7 vs. 17.2%; *p* = 0.01), non-significant higher risk of bleeding
Gunasakeran et al. (9)	317	All CAE phenotypes	All comers patients undergoing ICA	Long-term CV and survival outcomes at a mean f/u of 9.4 ± 1.8 years	Diffuse CAE and TIMI flow <3 are independent predictors of ACS (OR 4; 95% CI 2.0–7.8; *p* < 0.01); DAPT (17 vs. 34%; *p* = 0.03) or OAT (29 vs. 42%; *p* = 0.02) reduce the risk of ACS
Schram et al. (79)	77	All CAE phenotypes	STEMI	CAE as independent predictor of no-reflow compared with 154 controls	CAE independent predictor of no-reflow (OR 13.9; 95% CI 4.7–41.2, *p* < 0.001)
Shanmugam et al. (70)	25	All CAE phenotypes	STEMI	MACE in patients with EIRA compared with 80 controls at a mean f/u of 36.6 ± 14.1 months	EIRA patients had higher long-term incidence of composite CV events (44.0 vs. 16.3%; *p* = 0.01)
Iannopollo et al. (80)	32	Only CAAs	STEMI	Composite of all-cause death and recurrent MI compared with 2,280 controls at 30 days and 1 year f/u	CAA as culprit lesion associated with death and recurrent MI (HR 2.24, 95% CI 1.02–5.39, *p* = 0.04) and with ST (HR 6.29, 95% CI 2.32–17.05, *p* < 0.001)
Doi et al. (8)	51	All CAE phenotypes	STEMI NSTEMI	MACE compared with 1,647 controls at a median f/u of 49 months (19–93)	CAE associated with higher risk of MACE (HR 4.94; 95% CI, 2.36–10.4; *p* < 0.001). No MACE in patients receiving OAT with %TTR ≥60%
Nuñez-Gil et al. (81)	256	Only CAAs	STEMI NSTEMI	MACE compared with 500 controls at median f/u of 52 months (27–84)	Higher mortality (HR 3.1; 95% CI: 1.8–5.6; *p* < 0.01) and MACE (HR 2.3; 95% CI: 1.4–3.8; *p* < 0.01) in patients with CAA
Ipek et al. (72)	99	All CAE phenotypes	STEMI	Short and long-term (1 year) outcomes compared with 1,556 controls	Higher rates of no-reflow in EIRA (13.1 vs. 5.4%, *p* = 0.004) in patients. Non-significant differences in mortality
Campanile et al. (82)	101	All CAE phenotypes	STEMI	Short and long-term (2 years f/u) MACE	MACE in 6.9% cases during hospitalization in 17.8% at 1 year, and in 38.5% at 2 years. 8.9% of patients had a ST.
Erden et al. (73)	31	All CAE phenotypes	STEMI	Recurrence of MACE at short and long-term f/u compared with 612 controls	EIRA is an independent predictor of adverse outcome (OR 0.197; 95% CI 0.062–0.633; *p* = 0.006)
Baman et al. (69)	276	Only CAAs	All comers patients undergoing ICA	Mortality at 5 years f/u compared with 550 controls	CAA associated with mortality (HR 1.56; 95% CI 1.01–2.41; *p* = 0.04)
Demopoulos et al. (18)	203	All CAE phenotypes	All comers patients undergoing ICA	MACE at 2 years f/u	CAE does not confer additional risk in patients with coexisting CAD
Swaye et al. (1)	978	All CAE phenotypes	All comers patients undergoing ICA	Survival at 5 years f/u compared with 15,249 controls	No difference in survival was noted between CAE/CAA patients and non-CAE/CAA ones.

*%TTR, percent time in target therapy range; ACS, acute coronary syndrome; AMI, acute myocardial infarction; CAA, coronary artery aneurysm; CABG, coronary artery bypass grafting; CAE, coronary artery ectasia; CCS, chronic coronary syndrome; DAPT, dual antiplatelet therapy; EIRA, ectatic infarct-related artery; f/u, follow-up; HR hazard ratio; ICA, invasive coronary angiography; MACCE, major adverse cardiovascular and cerebral events; MACE, major adverse cardiovascular events; MI, myocardial infarction; NSTEMI, non-ST-elevation myocardial infarction; OAC, oral anticoagulation; OR, odds ratio; PCI, percutaneous coronary intervention; ST, stent thrombosis; STEMI, ST-elevation myocardial infarction; TIMI, thrombolysis in myocardial infarction; UA, unstable angina*.

## Invasive and Non-invasive Therapy

The optimal management of patients with CAE is largely unknown. The uncertainties regarding the natural history of CAE and the absence of robust randomized and large-scale data complicate the resolution of this clinical conundrum. Moreover, the most relevant amount of the current evidence on the treatment of CAE has been extracted from cohorts of symptomatic patients, often with ACS as clinical presentation, but conversely a lack of data on asymptomatic patients with incidentally found CAE and no evidence of significant CAD exists. Possible treatment options include medical therapy, PCI, and surgery, but each of these strategies reserves technical and clinical challenges. In absence of specific recommendations, management strategies are still individually tailored according to clinical presentation, anatomical features, and procedural complexity.

**Figure 5:**
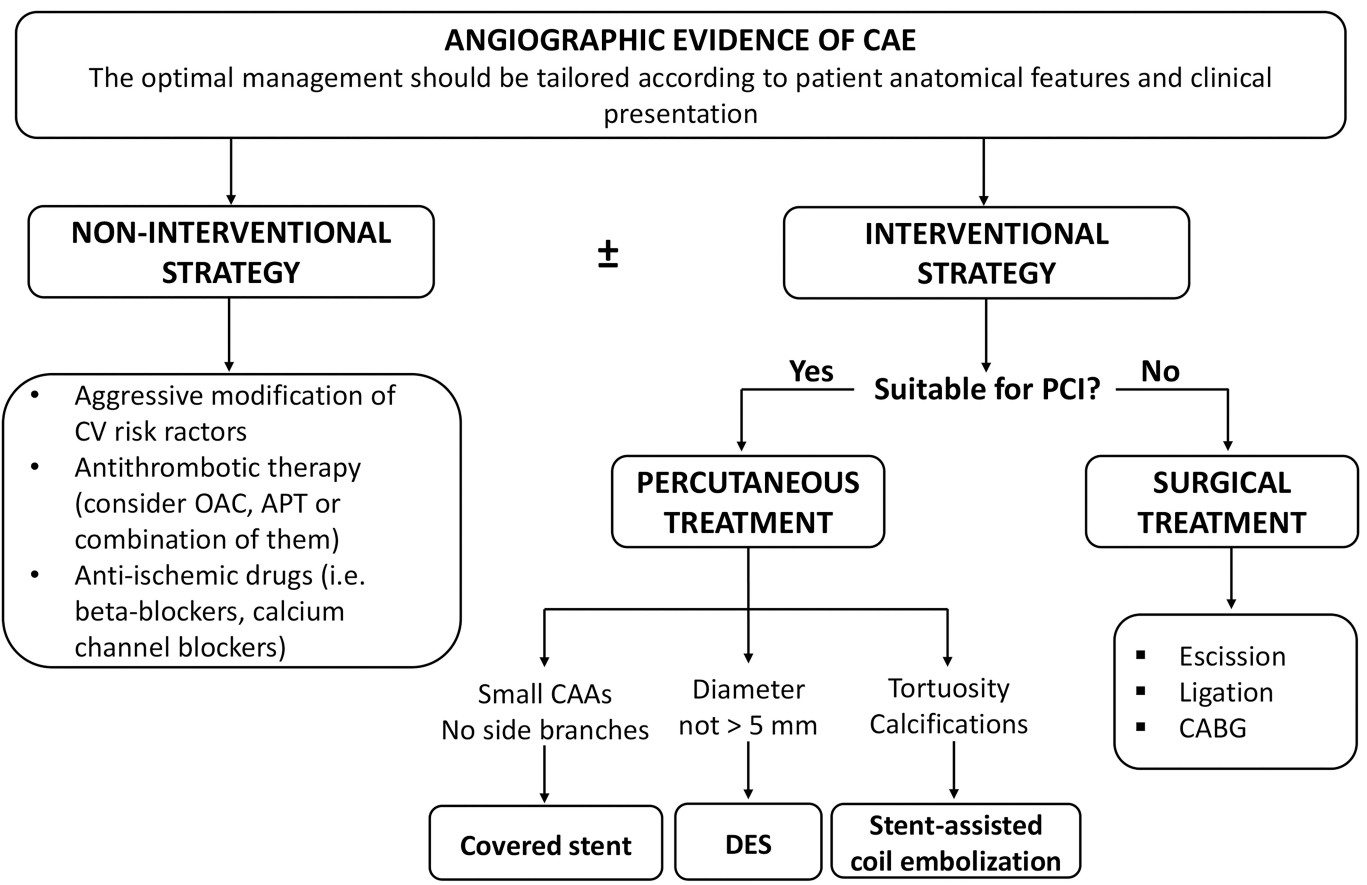
Clinical management of patients with CAE. APT, antiplatelet therapy; CAAs, coronary artery aneurysms; CABG, coronary artery bypass grafting; CAE, coronary artery ectasia; CV, cardiovascular; DES, drug-eluting stent; OAC, oral anticoagulation; PCI, percutaneous coronary intervention.

Figure 5 summarizes the clinical management of patients with CAE.

### Percutaneous Interventions

Percutaneous treatment of CAE is a valuable option in patients with suitable anatomical and clinical features. However, PCI of ectatic and aneurysmatic lesions presents several challenges, starting from lack of specific indications. First, it is uncertain whether conservative or interventional strategy is to be preferred in CAE patients without obstructive CAD, and there is no supportive evidence on PCI outcomes in this setting. Nonetheless, in patients with CAE and significant coronary stenoses and/or ACS, PCI has several technical challenges that must be carefully balanced into decision-making process when considering the optimal revascularization strategy. In the setting of an ACS with an EIRA, high thrombus burden with distal embolization and microvascular damage substantially increases the technical complexity of PCI, with a high risk of procedural failure and adverse events at long term (70). Given the high thrombus load, several reports showed a wider use of glycoprotein IIb/IIIa inhibitors and thrombus aspiration in patients treated with primary PCI of an EIRA (7, 72, 83). Despite these efforts, distal embolization with impairment of epicardial flow and/or microcirculatory reperfusion is frequently reported in these patients (73, 82). In a case-control study on 231 STEMI patients with and without CAE, Schram et al. found that CAE was an independent predictor of no-reflow after primary PCI (79). In selected cases of extensive thrombus burden and high risk of no-reflow, deferring stenting after an aggressive antithrombotic therapy may be an option. However, current evidence suggests that deferred stenting might be considered only in selected subsets of STEMI patients with high-risk anatomical features and to date there is no evidence in patients with CAE (84, 85).

Besides procedural complications, previous data showed that the presence of a CAA is a risk factor for stent thrombosis (ST) after PCI in patients with ACS (86). Iannopollo et al. assessed the 1-year clinical outcome of STEMI patients treated with primary PCI of a culprit lesion involving a CAA, and found that patients with an aneurysmal culprit had a higher recurrence of MI compared to those without, mainly driven by more definite ST (80). Several factors may explain the high risk of ST in CAE, including residual thrombus, flow disturbances and stent malapposition. Indeed, adequate stent sizing is one of the major challenges of PCI in CAE, even in non-ACS patients (87). Coronary angiography has some limitations in the visualization of aneurysmatic and ectatic segments in the deeper part of their lumen, especially in presence of endoluminal thrombotic material that might underestimate the true size of the vessel. Moreover, delayed antegrade contrast filling and dye stasis might further complicate the angiographic assessment of these lesions, and a prolongation of both contrast media injection and X-ray acquisition may be needed. Besides malapposition and thrombosis, inadequate stent sizing might also be associated with stent displacement and migration, especially in case of giant CAAs (87, 88). Assessment of lesion length and landing zone identification may also be difficult and often multiple overlapping stents are required to fully cover the diseased segments (89). The use of IVUS for PCI guidance appears to be mandatory to better define vessel architecture and provide accurate information for stent sizing and optimization, in order to reduce the risk of malapposition and ST (90). OCT may also be helpful, especially for a better assessment of thrombotic lesions and plaque morphology, but its application in CAE is limited by the incomplete blood clearance and low penetration depth in large vessels.

**Figure 6:**
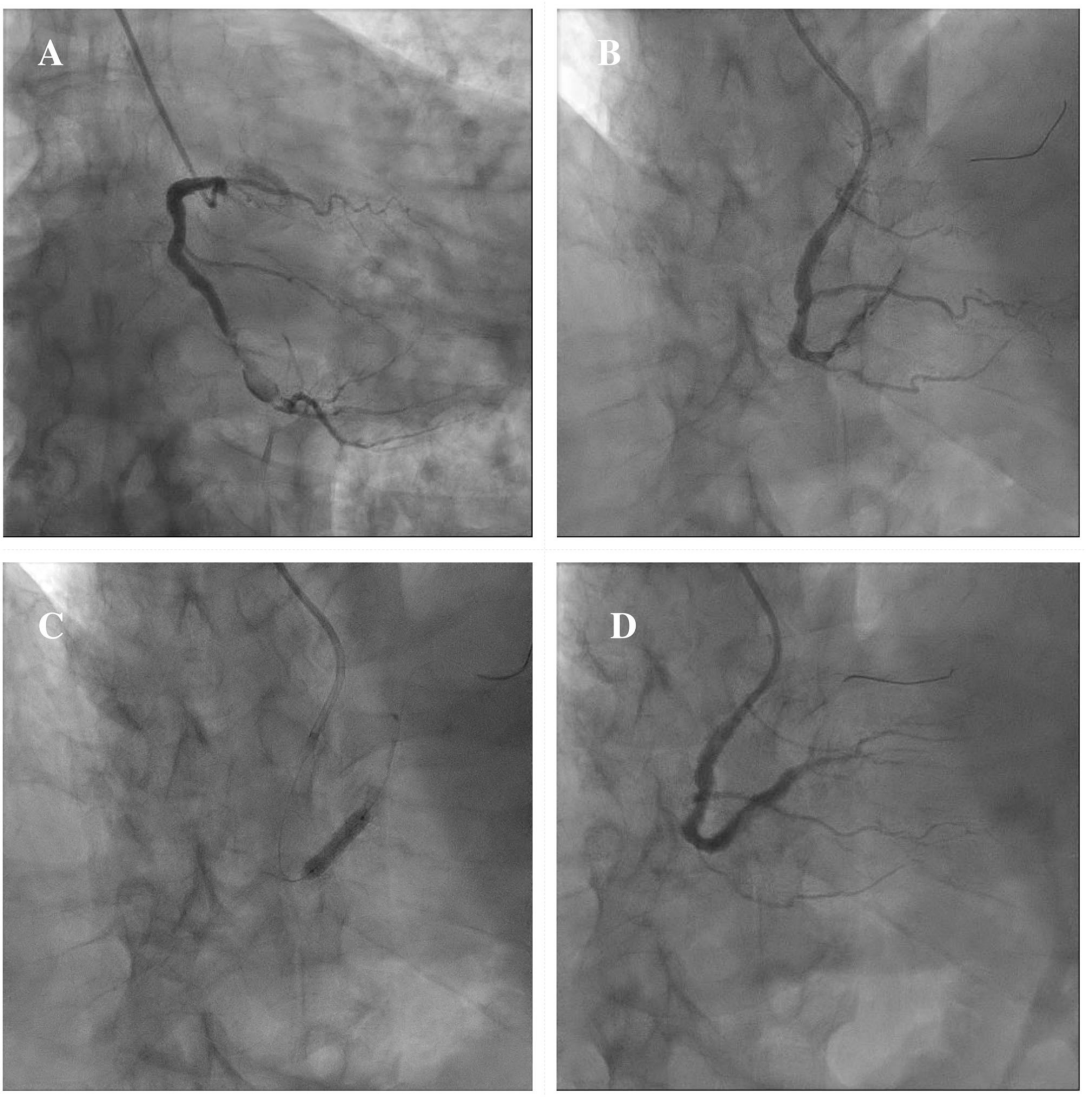
Example of exclusion of a saccular CAA in the distal RCA with high thrombus burden by means of covered stent implantation. Severe ISR of a previously implanted DES in the distal segment of the RCA continuing in a saccular CAA with high thrombus burden **(A)**. A 3.5 × 15 mm PK Papyrus covered stent is advanced to precisely seal the inlet and outlet of the CAA **(B)**. A DES Synergy (Boston Scientific, Marlborough, Massachusetts) 4 x 24 mm is then implanted proximally to the covered stent to treat the severe ISR **(C)**. Final result showing the exclusion of the saccular CAA **(D)**. CAA, coronary artery aneurysm; DES, drug-eluting stent; ISR, in-stent restenosis; RCA, right coronary artery.

In consideration of these technical challenges, if PCI is clinically indicated, different percutaneous techniques should be adapted to the anatomical scenario. Exclusion with covered stents might be indicated in saccular CAAs or small pseudoaneurysms not involving major side branches (11). Figure 6 illustrates a case showing the successful covered stent exclusion of a saccular CAA in the distal RCA with high thrombus burden. Different covered stents are currently available, but GRAFTMASTER (Abbott Vascular, Santa Clara, California) and PK Papyrus (Biotronik, Berlin, Germany) are the most frequently used. The GRAFTMASTER is constructed using a sandwich technique, with an ultra-thin layer of expandable polytetrafluoroethylene (PTFE) placed between 2 stainless steel stents, which are then pre-mounted on a balloon catheter delivery system. The stent can be used from diameters ranging from 2.8 to 5.5 mm and can be delivered through a 6 or 7 Fr guiding catheter, depending on the size of the device. PK Papyrus is a new-generation cobalt chromium single covered stent. Because of its advanced single stent design, it has greater bending flexibility and smaller crossing profile compared to GRAFTMASTER and can be delivered through a 5 or 6 Fr guiding catheter. For diameters from 5.5 mm to 10 mm the Atrium iCAST balloon expandable covered stent (MAQUET, Wayne, New Jersey) can be used, but an 8 or 9 Fr guiding catheter is needed. However, the use of covered stents is limited by several technical issues: the stiffness of the device, the poor deliverability and the need for large sheaths and guiding catheters are associated with a higher risk of procedural complications, especially in severely tortuous and calcified vessels. Moreover, the risk of side branch loss represents one the major limitation of covered stents. Different techniques have been described to avoid major side branches loss during covered stent implantation. GRAFTMASTER is a one-size stent mounted on different sized balloons and therefore its skirt can be shortened by ~3 mm with high-pressure post-dilatation using large non-compliant balloons: this technique allows to fully cover short CAAs, without landing at the level of major side branches (91, 92). Recently Davies et al. described a novel technique to treat large CAAs involving bifurcations with large side branches: first, a PK Papyrus is implanted from the proximal to the distal main vessel; second, a stiff guidewire with high tip load supported by a microcatheter is used to puncture across covered stent into the side branch; third, the microcatheter is advanced through the PK Papyrus to enlarge the opening and is used to exchange the stiff wire with a regular workhorse; Excimer laser atherectomy is then performed to ablate the polyurethane membrane of the covered stent at the level of the ostium of the side branch; finally, the PK Papyrus stent strut can be dilated with single balloon and double kissing balloon inflations (93). A similar fenestration technique of PK Papyrus without the use of excimer laser, has been carried out to gain access to the LAD in a case of covered stent implantation from the LM to the LCx for a perforation of the ostial LCx (94). However, these cases remain anecdotal and are not supported by data from observational studies; therefore, similar techniques should be limited to bail-out situations in specific anatomical settings and should be performed by experienced operators in complex PCI. Furthermore, in large CAAs involving major bifurcations, not suitable for percutaneous treatment, CABG remains a valuable option.

Beyond procedural technicalities, another limitation of covered stents is the high risk of ISR and ST at long-term follow up, with a substantial higher risk of target lesion revascularization (TLR) compared to new-generation DES (95–98). The underlying mechanisms are multifactorial and include impaired vascular healing, pro-thrombotic activation induced by the surface of stent material and neointimal proliferation at the edges of the covered stents caused by vessel trauma due to high-pressure balloon dilatations (99, 100). Recently Bossard et al. described a novel hybrid approach whereby the covered stent is “buried” with over-stenting and implantation of a new-generation DES, in order to benefit from its antiproliferative drugs and modern technology: the authors assessed the clinical outcome of 23 patients treated with this technique and showed low incidence of adverse events at long-term follow up, with low rate of TLR and no cases of ST (101). However, these results should be interpreted with caution because of the small number of patients evaluated, and should be confirmed in larger cohorts of patients treated with covered stent implantation.

**Figure 7:**
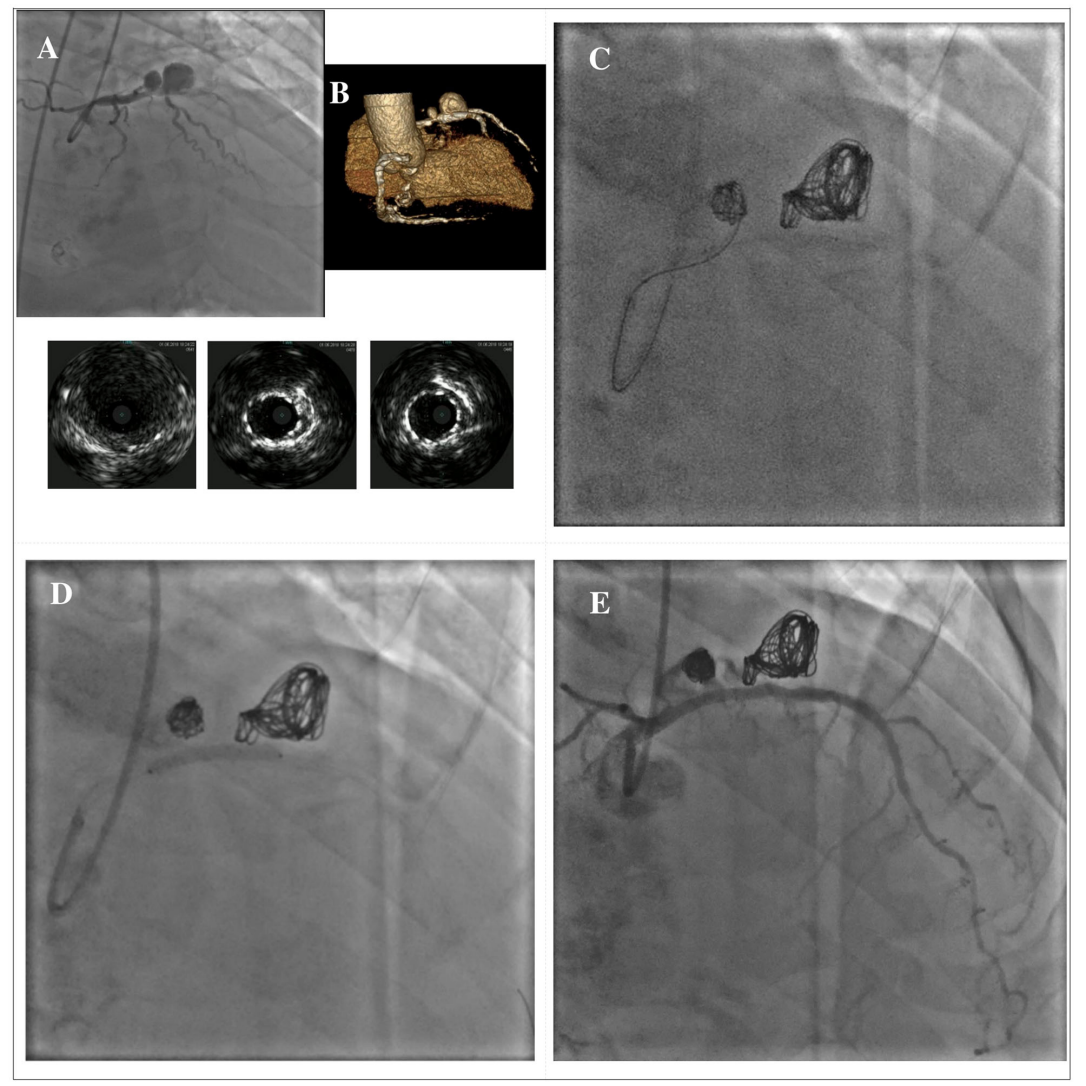
Hybrid approach for combined treatment with stent-assisted coil embolization and covered stent implantation in a dual-chamber iatrogenic pseudoaneurysm following PCI of the proximal LAD. The upper panel shows the angiographical view of an iatrogenic pseudoaneurysm of the proximal LAD due to a previous PCI with DES implantation; the lower panel shows the IVUS images of the pseudoaneurysm and its relationship with the previously implanted DES **(A)**. Rendered volume CCTA image showing a large dual-chamber pseudoaneurysm **(B)**. Multiple coils are advanced through a microcatheter to wrap around the old DES until completely filling both cavities of the pseudoaneurysm **(C)**. A BeGraft covered stent (Bentley InnoMed, Hechingen, Germany) 3 x 24 mm has been used to secure the coils correct positioning **(D)**. Final result **(E)**. ^*^Courtesy of Dr. Fabio Felice Tarantino. CCTA, coronary computed tomography angiography; DES, drug-eluting stent; IVUS, intravascular ultrasound; LAD, left anterior descending; PCI, percutaneous coronary intervention.

In cases where covered stent implantation is not possible due to severe tortuosity, calcifications or fear of side branch loss, stent-assisted coil embolization can be used, especially in large CAAs with wide neck. With this technique, commonly used in the treatment of cerebral aneurysms, a floppy guidewire supported by a microcatheter is directed inside the CAA before stent implantation. A coronary stent covering the entire neck of the CAA is then deployed at low pressure, jailing the microcatheter into the CAA; coils can then be passed through the microcatheter to wrap around the stent. Post-dilation of the stent is securely performed, after the microcatheter removal. Additional coils can be advanced through the stent struts if needed (102). There are several potential complications that should be considered during the procedure: the weakness of the aneurysmatic wall increases the risk of rupture during the embolization, therefore microcatheters, wires and coils should be manipulated with caution; another potential complication is coil herniation through the stent struts that may lead to acute or late stent thrombosis (103). Figure 7 illustrates a case showing a hybrid approach with stent-assisted coil embolization and covered stent implantation for the treatment of an iatrogenic dual-chamber pseudoaneurysm following PCI of the proximal LAD.

Percutaneous interventions are a valuable option for the treatment of SVGAs, especially in patients with high surgical risk and/or with preserved myocardial perfusion. These techniques have been increasingly used in recent years and include covered stent implantation, percutaneous closure with Amplatzer occluders and coil embolization with or without PCI of the native grafted vessel (57).

### Surgical Treatment

There is lack of robust evidence on the surgical treatment of CAE. Possible surgical techniques include CAA resection, ligation, or marsupialization with bypass grafting (104–106). However, there is uncertainty regarding the indications for surgery in patients with CAE. Khubber et al. in a single-center retrospective unadjusted study on 458 patients with CAA, found no significant differences in terms of adverse cardiovascular and cerebrovascular events between patients treated with CABG or PCI (77). Similar results were reported in 1,565 patients from the international coronary artery aneurysm registry (CAAR), with no differences in terms of adverse cardiovascular events and mortality between patients treated with either surgical or percutaneous approach (6). According to these findings, surgical treatment of CAE should be considered as the first-line therapy in cases where PCI is deemed to be at high risk due to complex anatomical features, like for CAAs involving the LM, multiple or giant CAAs, and SVGAs with graft thrombosis, impaired myocardial perfusion and acute mechanical complications (11, 57).

### Medical Therapy

The optimal medical therapy of patients with CAE is an area of ongoing debate. Considering that atherosclerosis is involved in the pathogenesis of CAE in most cases, an aggressive modification of cardiovascular risk factors should be mandatory in these patients.

However, antithrombotic therapy is the most controversial issue on the pharmacological treatment of CAE. Severe coronary dilatation is associated with flow disturbances and blood stasis, thus predisposing to the activation of coagulation cascade with increased risk of local thrombosis and distal embolization (18, 107). Moreover, an increased platelet reactivity has been described in patients with CAE (108). Given these pathophysiological considerations, oral anticoagulation (OAC) and prolonged dual antiplatelet therapy (DAPT) have been proposed as possible therapeutic strategies in these patients (11). However, literature provide only limited and conflictual evidence on this topic, especially in asymptomatic patients with incidentally found CAE. The need for a tailored pharmacological approach with aggressive antithrombotic regimens is justified by the growing evidence of a high risk of cardiovascular events in patients with CAE, especially in the setting of an ACS (7, 8, 74, 109). Doi et al. found that among 51 CAE patients with MI, those who received OAC and reached a time-in-target therapeutic range ≥60% did not experience major cardiovascular events at 49 months follow-up (8). Similarly, Gunasakeran et al. showed a lower incidence of recurrent ACS at long term in CAE patients treated with DAPT or OAC compared to those not receiving these treatments (9). Furthermore, in a propensity-matched analysis on 585 patients with CAAs from the CAAR registry, OAC significantly reduced the incidence of the composite endpoint of UA, MI and CAA thrombosis, with a non-significant increase in bleedings, mainly classified as Bleeding Academic Research Consortium type 1 (78).

However, because of the observational design and the relatively small sample size of these studies, these data should be interpreted with caution and should be confirmed by dedicated randomized trials or large multicentre registries.

In patients with KD OAC is recommended in selected patients with large and/or rapidly expanding CAAs (12). Moreover, intravenous administration of immunoglobulins in the acute phase is associated with high rates of CAA regression in these patients (42).

Given the role of atherosclerosis, inflammation and endothelial disfunction in the pathogenesis of CAE, statins and ACE-inhibitors have been proposed as possible therapeutic options due to their pleiotropic effects on the endothelium (110). Fan et al. evaluated the anti-inflammatory effects of rosuvastatin in patients with CAE, and found that levels of inflammatory biomarkers were significantly reduced after 6 months of treatment, especially in younger patients (111). Another study on 152 patients with CAE showed that polymorphism of the ACE gene, associated with a higher expression and activity of the enzyme, is independently associated with CAE (112). However, in absence of long-term studies, these data remain speculative.

Several anti-ischemic drugs have been proposed as possible therapeutic options in patients with CAE. Krüger et al. showed that the development of inducible ischemia in CAE depended on heart rate (5). Therefore, β-blockers (β-B) might be a reasonable option due to their negative chronotropic effect and reduction of myocardial oxygen demand in the absence of vasodilation (113). However, the use of β-B in patients with CAE has been questioned due to the risk of coronary spasm related to the unopposed α-receptors' stimulation (114).

Unlike β-B, calcium channel blockers (CCB) have anti-spastic effects and might have a beneficial role by reducing the risk of coronary spasm and thrombus formation (114). Other possible anti-ischemic effects of CCB in CAE include improvement of coronary blood flow and myocardial perfusion, and anti-hypertensive effects (18, 115).

Trimetazidine can also improve coronary flow and myocardial preconditioning by increasing adenosine levels, and showed to reduce exercise-induced myocardial ischemia in patients with CAE (116).

Unlike patients with CAD, nitrates should be avoided in patients with CAE because of their promotion of flow disturbances and exacerbation of myocardial ischemia (5).

## Gaps in Knowledge and Future Directions

Despite growing evidence on CAE in recent years, several controversies remain about its natural history and treatment. Although several studies showed a poor outcome in patients with ACS and angiographic evidence of CAE, the risk of future adverse events in asymptomatic patients with isolated CAE and non-obstructive CAD is unknown. Due to the absence of data from large multicenter studies and of specific recommendations, there is great uncertainty on what is the best medical and interventional treatment of isolated CAE.

In patients with obstructive CAD and indication for PCI, there are several procedural issues: (1) The use of intravascular imaging during PCI might be helpful for stent sizing and landing zone identification, but the large caliber may affect the quality of images, particularly for OCT; (2) The high thrombus burden in the setting of an ACS is associated with a higher risk of no-reflow, but the optimal periprocedural antithrombotic therapy has not been clarified yet; (3) The technology advances in interventional cardiology have provided several options for the percutaneous treatment of patients with CAE (i.e., DES, covered stents, coil embolization); however, the long-term performance of these devices needs to be investigated; (4) Optimal antithrombotic therapy is another missing piece in the complex framework of CAE; OAC and long-term DAPT have been proposed as possible therapeutic options, but the evidence is still controversial and limited to small number of patients.

Moreover, given atherosclerosis as the most prevalent underlying condition, it is unknown whether aggressive lipid lowering therapies with PCSK-9 inhibitors might provide a prognostic benefit in CAE patients with ACS. Lipoprotein (a) [Lp(a)], despite being associated with aortic and cerebral aneurysms, has an unclear role in the pathogenesis of CAE (117–119). In consideration of the association between high Lp(a) levels and adverse cardiac events in patients with CAD, Lp(a) might be a useful predictor for the prognostic stratification of CAE (120–122).

The renovated interest in CAE in the recent years emphasizes the crucial role of some required improvements in its knowledge pathway: the importance of a unique shared definition, of a deeper characterization of CAE phenotypes in real-world registries, and of effective therapies in randomized trials.

## Conclusions

CAE, defined as a diffuse or focal dilatation of an epicardial coronary artery, is reported in up to 5% of patients undergoing coronary angiography. The clinical manifestations of CAE are heterogeneous, ranging from asymptomatic cases to high-risk patients with ACS (17). There is growing evidence of an increased risk of adverse events at long term in patients with CAE, especially in those with ACS (7, 8). The management of these patients is challenging, and treatment options include optimal antithrombotic therapy, surgery, and percutaneous interventions with DES implantation, covered stent exclusion or stent-assisted coil embolization (11). Despite a deeper understanding of CAE in recent years, there are still come critical issues about is natural history and treatment that need to be address. Large multicenter studies are warranted to guide the clinician in the management of this complex setting of patients.

## Author Contributions

LE, MD, FC, and MB: drafting of the manuscript. AS, TA, FT, and GE: revising critically the manuscript. CB, CV, and GG: final approval of the manuscript. All authors contributed to the article and approved the submitted version.

## Conflict of Interest

The authors declare that the research was conducted in the absence of any commercial or financial relationships that could be construed as a potential conflict of interest.

## Publisher's Note

All claims expressed in this article are solely those of the authors and do not necessarily represent those of their affiliated organizations, or those of the publisher, the editors and the reviewers. Any product that may be evaluated in this article, or claim that may be made by its manufacturer, is not guaranteed or endorsed by the publisher.
